# Selpercatinib for treating recurrent mixed medullary and follicular cell-derived thyroid carcinoma: a case report

**DOI:** 10.1186/s40792-024-01898-7

**Published:** 2024-04-22

**Authors:** Mei Kadoya, Nobuyasu Suganuma, Yuka Matsubara, Hiroki Takase, Eita Kumagai, Soji Toda, Haruhiko Yamazaki, Katsuhiko Masudo, Satoshi Fujii, Aya Saito

**Affiliations:** 1 Department of Surgery, Yokohama City University School of Medicine, 3-9 Fukuura, Kanazawa-Ku, Yokohama, Kanagawa 236-0004 Japan; 2 Department of Molecular Pathology, Yokohama City University Graduate School of Medicine, 3-9 Fukuura, Kanazawa-Ku, Yokohama, Kanagawa 236-0004 Japan; 3 Department of Pathology, Yokohama City University Medical Center, 4-57 Urafunecho, Minami-Ku, Yokohama, Kanagawa 232-0024 Japan; 4 Department of Breast and Thyroid Surgery, Yokohama City University Medical Center, 4-57 Urafunecho, Minami-Ku, Yokohama, Kanagawa 232-0024 Japan; 5 Department of Endocrine Surgery, Kanagawa Cancer Center, 2-3-2 Nakao, Asahi-Ku, Yokohama, Kanagawa 241-8515 Japan

**Keywords:** Mixed medullary and follicular cell-derived thyroid carcinoma, *RET* mutation, Selpercatinib

## Abstract

**Background:**

Mixed medullary and follicular cell-derived thyroid carcinoma (MMFCC) is characterized by the coexistence of follicular and C cell–derived tumour cell populations within the same lesion. Due to its rarity, its etiology and clinical course remain unclear, and treatment for advanced or recurrent cases has not been established.

**Case presentation:**

We report a case of MMFCC treated with selpercatinib. The patient was a 69-year-old male presenting with tumors in the right thyroid lobe and in the upper mediastinum. Fine-needle aspiration (FNA) cytology of the right thyroid lobe tumor revealed a medullary carcinoma; germline *RET* mutations were not detected. After resection of the right thyroid lobe with central node dissection, rapid intraoperative diagnosis of the mediastinal mass confirmed malignancy, leading to total thyroidectomy with excision of the upper mediastinal tumor. Histologically, the tumor in the right thyroid lobe and the pretracheal lymph node revealed a mixture of medullary and follicular carcinoma components, diagnosed as MMFCC. The mediastinal lymph node exhibited only medullary carcinoma components. At 11 months postoperatively, computed tomography scans showed enlargement of the right supraclavicular and upper mediastinal lymph nodes. FNA cytology of the right supraclavicular lymph node suggested the recurrence of medullary thyroid carcinoma. The gene panel testing (The Oncomine Dx Target Test Multi-CDx system®, Thermo Fisher SCIENTIFIC) of metastatic lymph node revealed *RET* somatic mutation (M918T). Treatment with selpercatinib was initiated, and both the cervical and mediastinal lymph nodes showed a reduction in size.

**Conclusions:**

We report a rare case of selpercatinib use for MMFCC. Since *RET* mutations may occur frequently in MMFCC, selpercatinib could be effective in treating MMFCC.

## Background

Mixed medullary and follicular cell-derived thyroid carcinoma (MMFCC) is characterized by the coexistence of tumor components that differentiate into C cells and follicular epithelium within the same neoplasm. Because it is extremely rare, accounting for only 0.15% of all thyroid cancers [1], there are many uncertainties regarding its etiology and clinical course. There are few reported cases of treatments for advanced or recurrent MMFCC and no established therapies. Herein, we report a case of MMFCC that showed a favorable response to treatment with selpercatinib.

## Case presentation

**Fig. 1:**
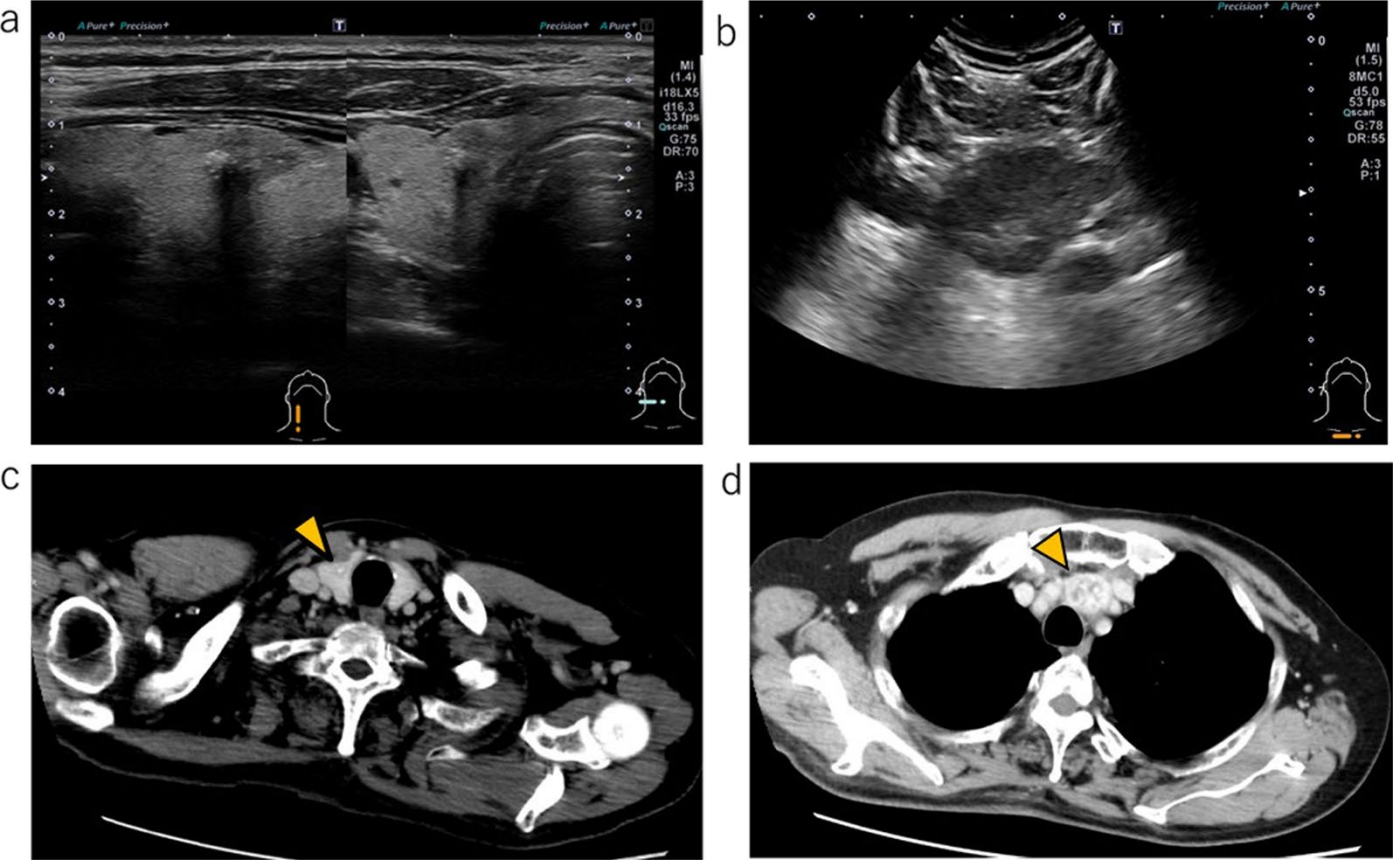
Preoperative ultrasonography and computed tomography (CT) scan. Ultrasonography revealed a hypoechoic nodule with internal calcification in the right thyroid lobe (**a**) and a hypoechoic mass in the superior mediastinum (**b**). CT scan showed a solid tumor with internal calcification in the right thyroid lobe (**c**) and a solid tumor with contrast enhancement in the superior mediastinum (**d**)

The patient was a 69-year-old male, referred to our hospital with a right lobe thyroid mass and mediastinal mass. He had no family history of thyroid cancer or radiation exposure. His anamnesis revealed stroke, angina pectoris, hypertension, and dyslipidemia. Physical examination revealed no mass in the neck. Cervical ultrasonography revealed a 12-mm hypoechoic nodule with internal calcification in the right lobe of the thyroid and a 40-mm hypoechoic mass in the superior mediastinum. Computed tomography (CT) scan showed an 11-mm solid tumor with internal calcification in the right thyroid lobe and a 40-mm solid tumor with contrast enhancement in the superior mediastinum, without evidence of distant metastasis (Fig. 1). Fine-needle aspiration (FNA) cytology of the right thyroid lobe nodule showed loose cohesiveness to isolated tumor cells with varying nuclear sizes, coarse granular chromatin with focal distribution, and relatively abundant cytoplasm. Amyloid-like substances were observed in the background, leading to the diagnosis of medullary thyroid carcinoma (MTC). Due to the difficulty in performing ultrasound-guided biopsy, the mediastinal tumor could not undergo cytological analysis. Blood tests showed elevated calcitonin (1036 pg/mL, normal: < 5.15 pg/mL) and carcinoembryonic antigen (CEA) levels (48.2 ng/mL, normal: 0.6–3.8 ng/mL). thyroid stimulating hormone (TSH), free triiodothyronine (FT3), free thyroxine (FT4), thyroglobulin, and thyroglobulin antibodies were within normal limits. Due to the cytological results and elevated calcitonin and CEA, the diagnosis of MTC was made. The mediastinal tumor was suspected to be either ectopic thyroid tissue or lymph node metastasis from MTC. Thus, surgical plan included intraoperative rapid diagnosis of the mediastinal tumor to determine the extent of thyroidectomy. Germline *RET* mutations were not detected.

The patient initially underwent right lobe thyroidectomy with central node dissection, but intraoperative biopsy of the upper mediastinal mass confirmed the presence of malignancy. Therefore, total thyroidectomy and open mediastinal tumor resection were performed.

**Fig. 2:**
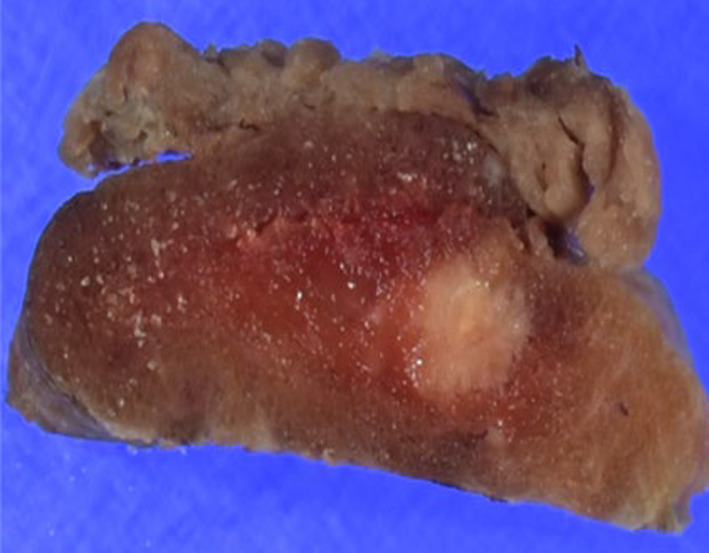
Macroscopic appearance of the right thyroid lobe mass. A 9 × 6 × 6 mm grayish-white tumor was identified in the right thyroid lobe

**Fig. 3:**
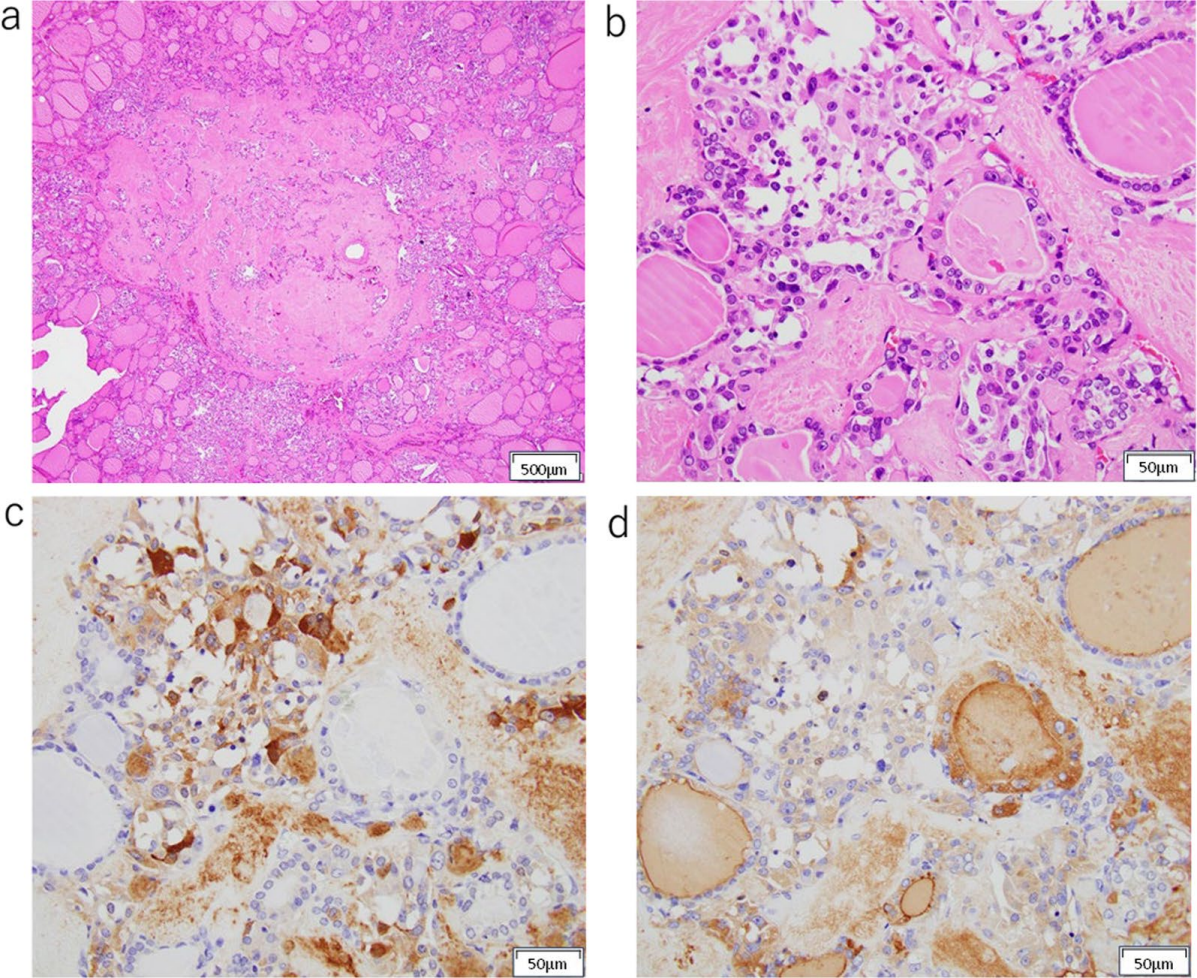
Microscopic examination of the right lobe thyroid mass. The tumor was mixture of two components: one component showed features of medullary thyroid carcinoma with positive immunostaining for calcitonin and negative for thyroglobulin, whereas the other component resembled follicular structures with positive staining for thyroglobulin and negative staining for calcitonin. **a** H&E × 40, **b** H&E × 400, **c** Calcitonin, **d** Thyroglobulin

**Fig. 4:**
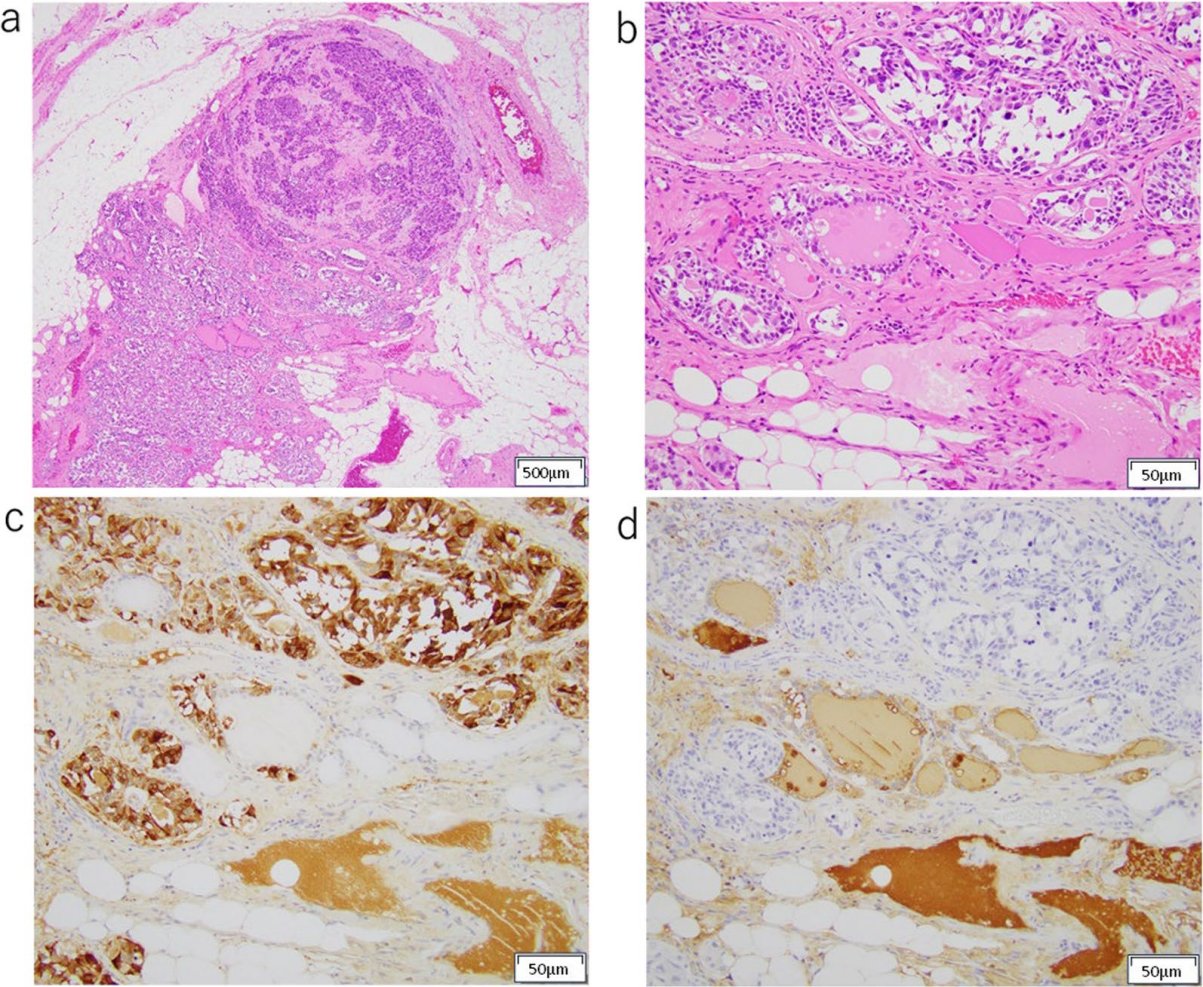
Microscopic examination of peritracheal lymph node metastasis. Immunohistochemical findings of peritracheal lymph node metastasis with mixed medullary and follicular cell-derived thyroid carcinoma: **a** H&E × 40, **b** H&E × 200, **c** Calcitonin, **d** Thyroglobulin

Macroscopically, a 9 × 6 × 6 mm partially indistinct grayish-white tumor was identified in the right thyroid lobe (Fig. 2). The mediastinal tumor measured 45 × 24 × 21 mm and appeared yellowish-white to grayish-white in color. Microscopically, the right lobe thyroid mass and peritracheal lymph node metastasis had two coexisting components. One component showed features of MTC, such as nests of short spindle-shaped or polygonal cells with nuclear atypia and eosinophilic granular cytoplasm with amyloid deposition in the background. The other component resembled follicular structures composed of glandular epithelial cells with swollen nuclei and distinct nucleoli. Immunohistochemical analysis revealed that the MTC component was positive for calcitonin and CEA and negative for thyroglobulin, whereas the follicular component was positive for thyroglobulin and negative for calcitonin and CEA (Figs. 3, 4). A final diagnosis of MMFCC was made. The mediastinal lymph node showed only medullary carcinoma components.

**Fig. 5:**
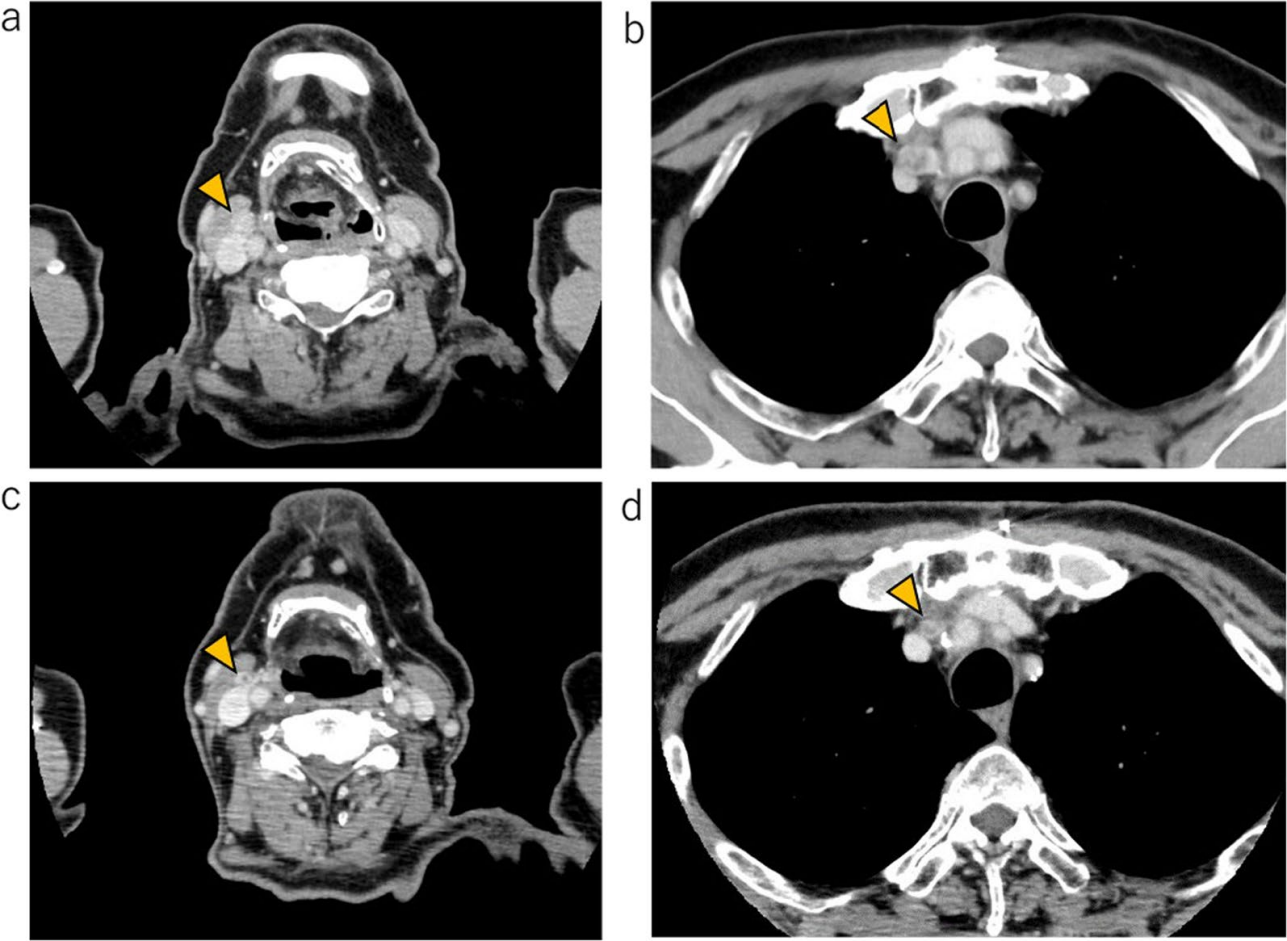
Computed tomography (CT) scans before and after selpercatinib treatment. CT scans at 11 months postoperatively revealed recurrence of mixed medullary and follicular cell-derived thyroid carcinoma at the right cervical lymph nodes (**a**) and upper mediastinal lymph nodes (**b**). After 9 weeks of treatment with selpercatinib, the cervical (**c**) and mediastinal lymph nodes (**d**) showed a reduction in size

**Fig. 6:**
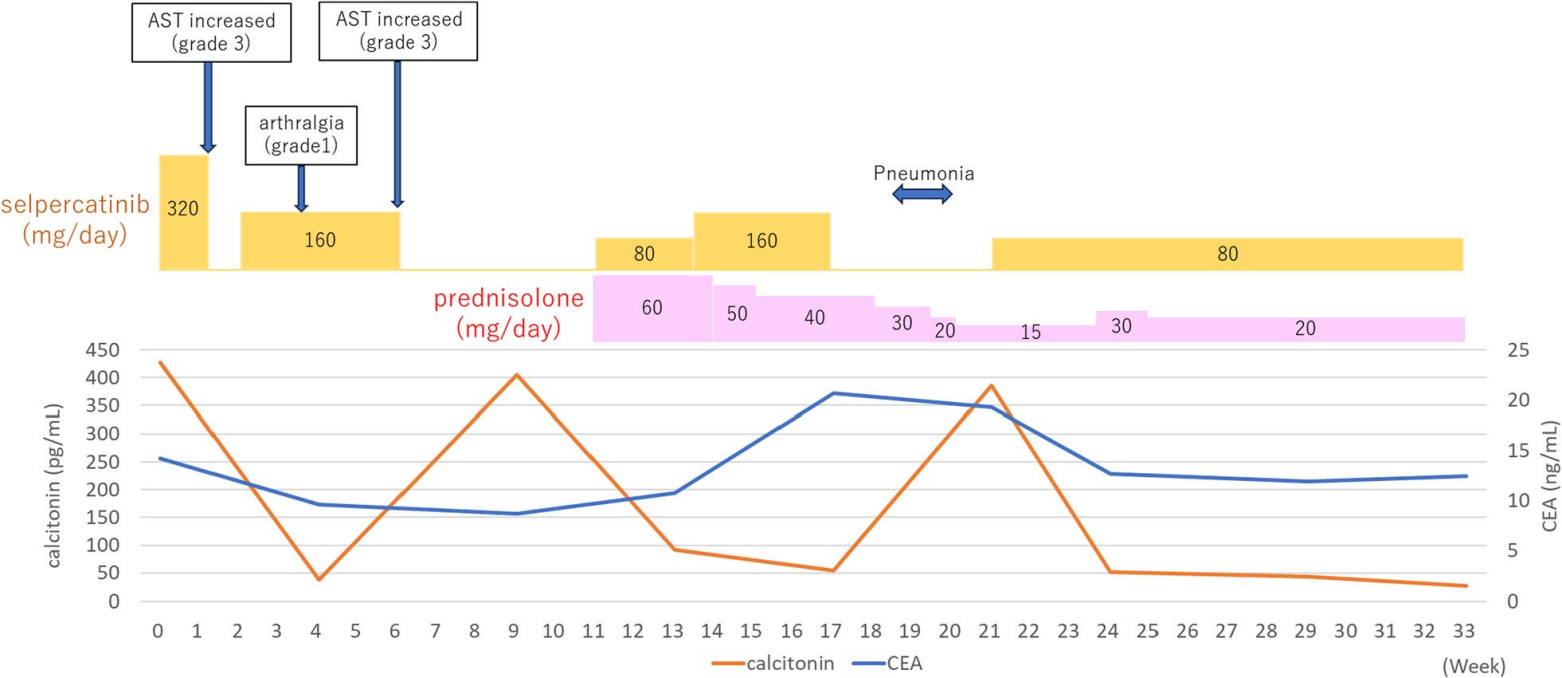
Treatment and clinical course after initiation of selpercatinib

Postoperatively, thyroglobulin levels remained below the measurable sensitivity, whereas calcitonin and CEA levels remained elevated. At 11 months postoperatively, CT scans revealed enlargement of the right cervical and upper mediastinal lymph nodes (Fig. 5a, b). FNA cytology of the right cervical lymph node confirmed metastasis of the MTC. The Oncomine Dx Target Test system, a gene panel test using a next-generation sequencer, of mediastinal lymph node metastasis was positive for *RET* somatic mutation (M918T). At 15 months postoperatively, treatment with selpercatinib at a dose of 320 mg/day was initiated. However, after 5 days, aspartate aminotransferase (AST) increased (grade 3, Common Terminology Criteria for Adverse Events version 5.0) was observed, which led to temporary discontinuation and subsequent resumption at a reduced dose of 160 mg/day. Because of the recurrence of AST increased (grade3) and development of arthralgia (grade1), the dose was further reduced to 80 mg/day, and prednisolone at 60 mg/day was initiated to consider the possibility of hypersensitivity reaction. After 18 weeks of treatment, the patient developed bacterial pneumonia and discontinued selpercatinib for 4 weeks, after which it was resumed (Fig. 6). At 9 weeks after initiation, both the cervical and mediastinal lymph nodes showed a reduction in size, indicating a partial response, which was maintained at 33 weeks after initiation (Fig. 5c, d).

## Discussion

In 1988, the World Health Organization defined MMFCC as a tumor that displays the morphological features of both medullary and follicular carcinomas with immunoreactivity for calcitonin and thyroglobulin, respectively [2]. The follicular cell–derived component includes papillary thyroid carcinoma, follicular, oncocytic, poorly differentiated, or anaplastic carcinoma. The etiology of MMFCC is not well understood, and several theories have been reported [3]: ① differentiation from a common stem cell into two tumors (stem cell theory), ② differentiation of medullary carcinoma into follicular epithelium (divergent differentiation theory), ③ simultaneous carcinogenic stimuli affecting follicular epithelium and C cells in a particular area (field effect theory), ④ collision between separately developed follicular epithelial tumors and medullary carcinoma (collision effect theory), and ⑤ incorporation of normal follicular cells into medullary carcinoma leading to tumorigenesis (hostage theory).

The diagnosis is not difficult if the follicular component is a papillary carcinoma, because it has characteristic nuclear findings and structure. However, in cases of follicular carcinoma, caution is needed even if follicular structures are observed within the medullary carcinoma, as these may represent normal follicles incorporated into the medullary carcinoma. In our case, the follicular structure was composed of glandular epithelial cells with nuclear enlargement, distinct nucleoli, and coarse chromatin, which differed from normal follicles.

In certain studies, *RET* mutations have been reported in MMFCC [4]. Cases associated with multiple endocrine neoplasia 2A (MEN2A) and multiple endocrine neoplasia 2B (MEN2B) [5, 6] and a case in which a somatic lineage *RET* mutation [3] have also been reported. In this study, the preoperative germline *RET* mutation was negative; whereas the somatic *RET* mutation was positive. Volante et al. reported that somatic *RET* gene mutations were found in the medullary carcinoma component but not in the follicular cell component [3], and thus, the site of examination may be important when testing for somatic *RET* gene mutations. In our case, cytology of the recurrent lymph node revealed pathological findings of medullary carcinoma, and CEA and calcitonin levels were high and thyroglobulin levels were low in the wash-out fluid from FNA of the recurrent lymph node. Based on these findings, we concluded that the recurrent lesion had a medullary carcinoma component, which should be the primary target for treatment. Therefore, the gene panel testing of mediastinal lymph node metastasis with a major proportion of medullary carcinoma components was performed to determine the *RET* gene somatic mutations.

The clinical outcomes of MMFCC are closely associated with the predominance of MTC components, and surgery is the first-line treatment for MMFCC as well as MTC [7]. The ATA and NCCN guidelines suggest that total thyroidectomy with central lymph node dissection is the standard treatment for MTC, and it is not clearly defined to what extent a lobectomy is acceptable [8, 9]. In our case, the primary tumor was located in the right lobe of the thyroid gland, but mediastinal lymph node metastasis was on the left side. Furthermore, the mediastinal lymph node metastasis was accompanied by extra-nodal invasion, so a total thyroidectomy was performed to ensure central lymph node dissection. There are reports of radioactive iodine therapy being administered due to the follicular cell-derived components [7, 10, 11]; its efficacy has not been clarified. A high rate of lymph node metastasis has been observed, and distant metastasis to the lung, liver, and bone has also been reported [12, 13]. Notably, therapy for patients with advanced or recurrent MMFCC has not yet been established. In a study by Liu et al. that used surveillance, epidemiology, and end results (SEER) data from 2000 to 2020, chemotherapy was used to treat four patients with MMFCC, but the details are unclear [14]. In our case, due to the risk associated with repeat surgery for the mediastinal lymph node recurrence, we decided to initiate treatment with selpercatinib, a *RET* inhibitor. Selpercatinib selectively inhibits activated *RET*, thereby inhibiting tumor growth, and it has demonstrated high efficacy and safety in patients with *RET* mutation-positive MTC in the LIBRETTO-001 trial [15]. In addition, selpercatinib had superior therapeutic effects versus other multikinase inhibitors (cabozantinib and vandetanib) in patients with *RET* mutation-positive MTC as shown in the LIBRETTO-531 trial, a multicenter, nonblinded, randomized phase III trial [16]. Furthermore, it has been suggested that changes in the tumor microenvironment caused by prior VEGF-targeted therapies may result in resistance to RET inhibitors [17]. For these reasons, selpercatinib was selected as the treatment of choice in this case. Nevertheless, there are no other reported cases of selpercatinib use for MMFCC other than this case; the accumulation of cases in the future is necessary to confirm our findings. Microdissection of medullary carcinoma components and follicular cell-derived components followed by analysis using next-generation sequencing may lead to an understanding of the etiology, diagnosis, and treatment of MMFCC.

## Conclusions

We report a first case of MMFCC with somatic *RET* mutation which was successfully treated with selpercatinib. Since *RET* mutations may occur frequently in MMFCC, further studies are required to determine the frequency of mutations and the efficacy of *RET* inhibitors.

## Data Availability

All data generated or analyzed during this study are included in this published article.

## Abbreviations

MMFCC: Mixed medullary and follicular cell-derived thyroid carcinoma
CT: Computed tomography
FNA: Fine-needle aspiration
MTC: Medullary thyroid carcinoma
CEA: Carcinoembryonic antigen
TSH: Thyroid stimulating hormone)
FT3: Free triiodothyronine
FT4: Free thyroxine
AST: Aspartate aminotransferase
MEN2A: Multiple endocrine neoplasia 2A
MEN2B: Multiple endocrine neoplasia 2B
ATA: American Thyroid Association
NCCN: National Comprehensive Cancer Network
SEER: Surveillance, epidemiology, and end results
